# Résultats fonctionnels de la chirurgie de la cataracte par phacoalternative avec implantation en chambre postérieure: à propos de 300 cas à Bobo Dioulasso (Burkina Faso)

**DOI:** 10.11604/pamj.2015.20.230.6323

**Published:** 2015-03-12

**Authors:** Jean Wenceslas Diallo, Nonfounikoun Meda, Ahgbatouhabéba Ahnoux-Zabsonre, Claudette Yameogo, Mariam Dolo, Jérôme Sanou, Arsène Daboue

**Affiliations:** 1 Centre Hospitalier Universitaire Sourô Sanou Bobo, Dioulasso, Burkina Faso; 2 Centre Hospitalier Universitaire Yalgado Ouédraogo, Ouagadougou, Burkina Faso

**Keywords:** Chirurgie, cataracte, phacoalternative, Burkina Faso, surgery, cataract, alternative to phaco, Burkina Faso

## Abstract

La cataracte est la première cause de cécité curable dans le monde. Son traitement est chirurgical. Le but de notre travail a été d’évaluer les résultats de la phacoalternative ou la chirurgie de la cataracte à petite incision. Il s'est agi d'une étude transversale descriptive à collecte prospective allant du 1er janvier au 31 septembre 2014, chez des patients âgés d'au moins 40 ans. Les données socio-démographiques, l'acuité visuelle, l'astigmatisme et les complications ont été évalués. Nous avons inclus 300 yeux de 286 patients. L’âge moyen était de 66 ans (écart type 9,93) avec une prédominance masculine de 57,7%. Les co-morbidités étaient dominées par l'hypertension artérielle 30,33% des cas. L'acuité visuelle pré-opératoire était de moins de 1/20è dans 70, 7% des cas. En biométrie, la puissance moyenne était de 21,50 dioptries. L'implant posé a été adéquat dans 60%. Les principales complications per-opératoires étaient le chémosis post-anesthésie 4,67% et l'issue de vitrée moins de 2% des cas. Les complications post opératoires précoces ont été dominées par l’œdème de cornée 26,33%, et les complications tardives par la cataracte secondaire. L'astigmatisme induit était de 1, 12 dioptrie en moyenne (écart type 1,26). Sans correction, les résultats visuels étaient mauvais dans moins de 1%, limites dans 31%, et bons 68% suivants les normes de l'Organisation Mondiale de la Santé. La phacoalternative donne des résultats satisfaisants, avec peu de complications. L'amélioration du plateau technique et la disponibilité d'implants adéquats pourraient les améliorer.

## Introduction

La cataracte est la première cause de cécité curable dans le monde et représente 50% de l'ensemble des causes de cécité [1]. En Afrique subsaharienne la prévalence de la cécité est estimée à 1,4% [2]. Au Burkina-Faso le taux de cécité par cataracte est estimé à 65% [3]. Le traitement de la cataracte est chirurgical, plusieurs techniques existent dont la référence est la phocoémulsification. Cependant, sa pratique est peu répandue dans les pays en développement du fait de son coût. La phacoalternative ou chirurgie de la cataracte à petite incision (ou Small Incision for Cataract Surgery SICS) donne des résultats comparables à la phacoémulsification [4]. Au Burkina-Faso, Méda a rapporté 89,2% de bons résultats visuels avec correction par la technique de l'extraction extra capsulaire [5]. La phacoalternative tend à supplanter l'extraction extra capsulaire et à devenir la technique de référence dans les pays en voie de développement. Elle est de pratique relativement récente dans notre pays. C'est pourquoi nous nous proposons d’évaluer les résultats fonctionnels de cette technique et contribuer ainsi à la prise en charge de la cataracte.

## Méthodes

Nous avons mené une étude transversale descriptive à collecte prospective du 1^er^janvier 2014 au 30 septembre 2014 à la clinique d'ophtalmologie située dans le camp militaire Ouézzin Coulibaly de Bobo Dioulasso. Cette clinique est un service de la direction régionale du service de santé des Armées de la 2^ème^ région militaire. Les patients âgés d'au moins 40 ans, présentant une cataracte et consentant ont été inclus. Nous avons exclu de notre étude les patients présentant une cataracte post-traumatique, ou une neuropathie optique connue ou un syndrome drépanocytaire majeur. L’échantillonnage a été exhaustif incluant tous les patients répondant aux critères d'inclusion et vus au cours de notre période d’étude. Une fiche d'enquête a été élaborée et renseignée à partir des dossiers et par interview des patients. Nous avons décrit les variables en rapport avec les caractéristiques socio démographiques, les antécédents médicaux, les données de l'examen clinique pré opératoire, les complications per et post opératoires, l'acuité visuelle post opératoire, l'astigmatisme post opératoire et induit. L'anesthésie a été péribulbaire. Tous les patients ont été opérés par le même chirurgien dans les mêmes conditions avec une implantation en chambre postérieure (ICP). Nous avons utilisé des implants en polyméthylmétacrylate (PMMA). L'incision a été faite en supérieur lorsque l'astigmatisme pré opératoire était directe, et en temporal lorsqu'il était inverse. Les patients ont été vus en post opératoire le1er jour (J1), puis le 15^ème^(J15), 30^ème^(J30), et 60^ème^ jour (J60). La saisie des données a été faite sur EPI-Info version 7 et l'analyse grâce au logiciel Stata. Les tableaux ont été faits sur Excel 2007. Le test de Chi2 de Pearson ou le test de Fisher ont été utilisés pour la comparaison des proportions.

## Résultats

Au cours de notre période d'inclusion 384 yeux ont été opérés de la cataracte à la clinique d'ophtalmologie, parmi lesquels nous avons inclus 300 yeux de 286 patients.

### Résultats pré opératoires

**Figure 1:**
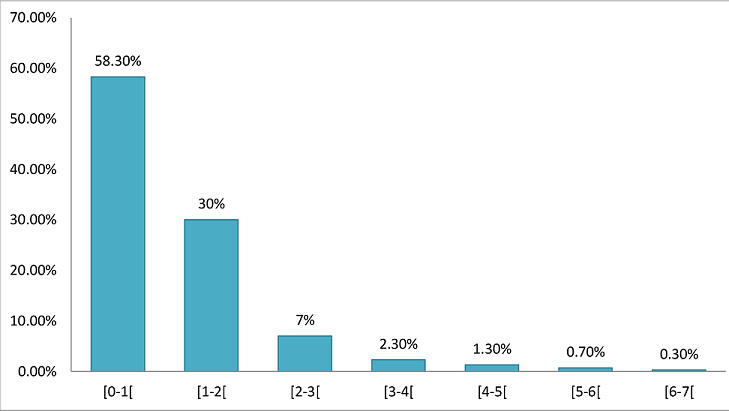
Répartition des cas selon l'astigmatisme pré-opératoire (n = 300)

**Figure 2:**
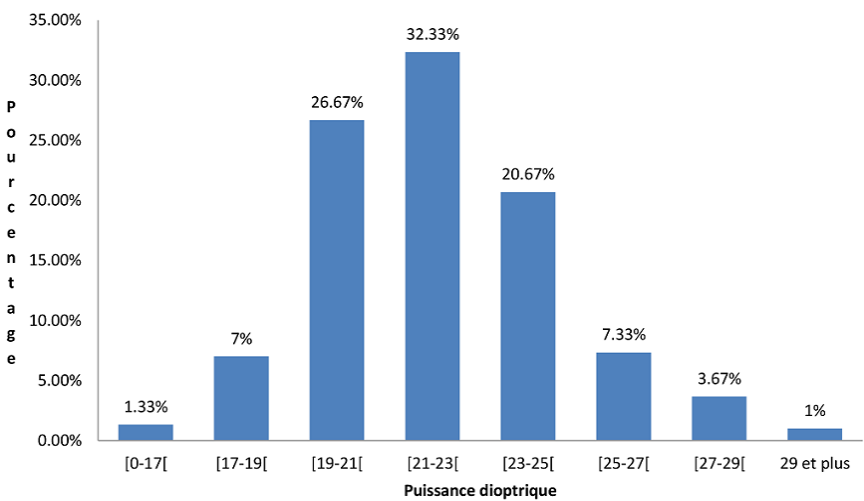
Répartition des cas selon la biométrie en pré opératoire (n = 300)

L’âge moyen de nos patients était de 66 ans (écart type de 9,93) avec des extrêmes de 40 et 93 ans. La tranche d’âge la plus représentée était celle de 60 à 69 ans avec 110 cas soit 36,70%. Il y avait 173 hommes (57,7%) et 127 femmes (42,3%) soitun sex-ratio de 1,36. Les patients résidaient à Bobo en majorité avec 177 cas soit 59%. Dans 56% des cas ils n’étaient pas scolarisés, et les professions les plus représentées étaient les femmes au foyer 34,7% des cas, suivit des cultivateurs 21,3%. Chez 120 patients (40%) une pathologie générale était associée à la cataracte, et la plus fréquente était l'hypertension artérielle avec 91 cas (30,33%) suivie du diabète 2,33%. L'acuité visuelle pré opératoire était inférieure à 1/20 dans 212 cas (70,7%), et 74 yeux (24,7%) avaient une acuité comprise entre 1/20 et 3/10. Nous avons noté 10 cas (3,33%) de dystrophie cornéenne, une hypertonie dans 2% des cas. Le fond d’œil était inaccessible dans 243 cas soit 81%. L'astigmatisme pré-opératoire moyen était de 0,87 dioptrie (D) (écart type de 1,040) avec des extrêmes de 0 et 6,37 D (Figure 1). La classe modale est celle de 0 à 1D avec 175 cas (58,3%). La médiane de la biométrie était de 21,50 D (écart type de 2,70) et les extrêmes de 7 et 30,50 D. La classe la plus représentée était celle de (21 à 23D) avec 97 cas soit 32,33% (Figure 2).

### Résultats post opératoires

**Figure 3:**
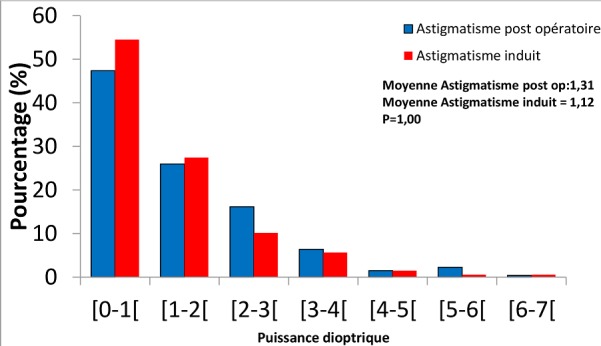
Répartition de l'astigmatisme post opératoire et l'astigmatisme induit (n = 266)

L’œil opéré a été le droit dans 170 cas soit 56,7%. Dans 179 cas (59,67%) l'implant correspondait à la puissance calculée par la biométrie, et chez 121 cas (40,3%) l'implant était inadéquat avec une différence allant de - 5 à + 4 D. La complication per-opératoire la plus fréquente a été le chémosis secondaire à l'anesthésie avec 4,67%. L'issu de vitrée et l'hémorragie sous conjonctivale ont été notées dans 1,33% des cas chacune. Les complications post opératoires précoces décrites dans le Tableau 1 étaient dominées par l’œdème de cornée avec 79 cas (26,33%) à J1, d’évolution favorable. Les autres complications étaient la kératite ponctuée superficielle 7,7% des cas et l'hyphéma dans 13 cas soit 4,3%. Les complications tardives sont décrites dans le Tableau 2. A J30 post-opératoire 286 patients (95,33%) ont été revus. Seuls 2 cas d’œdème de cornée persistaient. A J60 post opératoire, 225 patients sur 300 ont été revus soit 75%. On ne notait plus d’œdème de cornée, mais 6 cas d’érosion cornéenne superficielle qui ont eu une évolution favorable. La pupille était irrégulière dans 8% des yeux à J60, et une cataracte secondaire était notée dans 12 yeux (5.33%). L'astigmatisme post-opératoire a été mesuré chez 266 cas (88,67% des patients) à J33 (Figure 3). La moyenne était de 1,316 D (écart type de 1,69), et des extrêmes de 0 et 6,87 D. L'astigmatisme induit était en moyenne de 1,12 D avec des extrêmes de 0 et 6,87 D. Au plan fonctionnel, l'acuité visuelle de loin sans correction mesurée à J30 chez 286 patients montrait que chez 194 soit (67,83%) parmi eux elle était supérieure ou égale à 3/10ème, et à J60 cette proportion était de 74,22% (218 yeux) comme le montre le Tableau 3.

**Tableau 1:** Fréquence des complications post opératoires précoces à J1 (n = 300) et à J15 (n = 295)

Complications	J1 (n = 300)		J15 (n = 295)	
	Effectif	Pourcentage	Effectif	Pourcentage
Cornée	p = 0,0001			
Œdème de cornée	79	26,33%	9	3,05%
Kératite superficielle	23	7,66%	29	9,83%
Autre	1	3,33%	7	2,37%
Chambre antérieure	p = 0,0001			
Hypotahalamie	2	0,7%	2	0,68%
Tyndall	8	2,7%	3	1,01%
Hyphéma	13	4,3%	0	0%
Pupille	p = 0,0001			
Décentrée	1	0,3%	0	0%
Irrégulière	10	3,3%	13	4,40%
Mydriase	0	0%	46	15,60%
Myosis	22	7,3%	1	0,34%
Pression intraoculaire	p = 0,0001			
Hypertonie			28	9,49%
Implant	p = 0,0001			
Décentré	1	0,3%	5	1,7%
Masse résiduelle				
Présence	15	5%	9	3,05%

**Tableau 2:** Fréquence des complications post opératoires tardives à J30 (n = 286) et à J60 (n = 225)

Complications	J30 (n = 286)		J60 (n = 225)	
	Effectif	Pourcentage	Effectif	Pourcentage
**Cornée**	p = 0,0001			
Œdème de cornée	2	0,7%	0	0%
Kératite superficielle	11	3,85%	5	2,22%
Autre+	8	2,79%	6	2,67%
**Chambre antérieure**	p = 0,0001			
Hypothalamie	1	0,35%	0	0%
**Pupille**	p = 0,0001			
Irrégulière	21	7,34%	18	8%
Myosis	1	0,35%	0	0%
**Pression Intra-Oculaire**	p = 0,0001			
Hypertonie	5	1,75%	2	0,89%
**Implant**	p = 0,0001			
Décentré	4	1,4%	0	0%
**Masse Résiduelle**	p = 0,0001			
Présence	5	1,75%	3	1,33%
**Cataracte Secondaire**				
Oui	10	3,5%	12	5,33%

+ Kératite bulleuse, ulcère de cornée

**Tableau 3:** Distribution de l'acuité visuelle post opératoire selon les normes de l'OMS pour la chirurgie de la cataracte à J30 (n = 286) et J60 (n = 225)

Acuité visuelle	J30 post-opératoire (n = 286)				J60 post-opératoire (n = 225)			
	Acuité sans correction		Meilleure acuité visuelle		Acuité sans correction		Meilleure acuité visuelle	
	n	%	n	%	n	%	n	%
< 1/10	2	0,7%	2	0,7%	1	0,44%	1	0,44%
1/10-2/10	90	31,47%	14	4,9%	57	25,33%	6	2,67%
≥ 3/10	194	67,83%	270	94,4%	167	74,22%	218	96,89%

## Discussion

Notre étude présente certaines limites. La situation de la clinique d'ophtalmologie dans un camp militaire peut être un biais de sélection des patients. Toute fois le camp est situé au centre-ville et son accès est ouvert à toute la population pour les soins de santé. Ainsi presque tous nos patients étaient des civils. L'observance des patients aux rendez-vous pour le suivi a été dégressive au cours de la période d’étude. En effet de 100% à J1, le taux de patients vus en post opératoire est passé à 98,33% à J15, 95,33% à J30, et 75% à J60. Une amélioration de l'acuité visuelle chez certains patients peut les conduire à juger inutile de revenir, en particulier ceux qui résident en dehors de la ville de Bobo Dioulasso. Cet état peut avoir un impact sur les statistiques dans notre étude. Les implants adéquats par rapport à la biométrie n’étaient pas toujours disponibles. Dans ces cas nous avons utilisé des implants de puissance approximative. Ceci a pu induire une amétropie. Nous pensons néanmoins que, la collecte prospective des données, la mesure de la biométrie chez tous les patients inclus, la mesure de l'astigmatisme pré opératoire, post opératoire et induit constituent des points forts pour de notre étude.

La moyenne d’âge des patients est variable dans la littérature, suivant les critères d'inclusion. Nous avons inclus des patients d'au moins 40 ans et avons retrouvé une moyenne d’âge de 66 ans qui est proche de la littérature [6, 7]. Cette moyenne d’âge est plus élevée dans les pays occidentaux pour les cataractes séniles du fait de leur survenue plus tardive. Lorsque l'on considère les cataractes de toutes étiologies, les moyennes d’âge sont basses et varient de 56 à 60 ans [5–8] du fait de l'incidence des cataractes post traumatiques qui surviennent chez les sujets jeunes, et les cataractes congénitales. Nous avons retrouvé une prédominance masculine (57,7%) avec un sex-ratio de 1,36 comme c'est souvent le cas dans la littérature [5]. Cette prédominance masculine contraste avec les données démographiques du Burkina-Faso où les femmes représentent 52% de la population générale [9]. Le facteur économique pourrait être un frein à l'accès aux soins chez les femmes dans certaines régions. Dans d'autres régions la prédominance était plus tôt féminine [6]. Dans les antécédents, l'hypertension artérielle (30,33%) a été la principale co morbidité, très proche des résultats de Méda 31,7% [5]. L'acuité visuelle pré opératoire dans notre étude était inférieure à 1/20 chez 70,7% des patients. Cette proportion est plus basse que ceux de Meda [5] au Burkina-Faso qui trouvait 75%, ou Guirou [6] 93,6% au Mali. Ce tableau est caractéristique des pays en développement et pourrait s'expliquer par la consultation tardive des patients contrairement aux pays développés. L’œil majoritairement opéré était le droit avec 56,70% des cas, comme cela est souvent le cas dans la littérature. En pré opératoire, nous avons observé 3,33% de cas de dystrophie cornéenne. C'est la lésion la plus fréquente. En effet Meda [5] retrouvait 8,3%. Ces lésions sont secondaires aux atteintes cornéennes diverses par le trachome, les traumatismes, les ptérygions.

Dans notre étude l'astigmatisme pré-opératoire moyen était de 0,87D semblable à ce qui est rapporté par Baraquet [10] 0,78 D, Briesen [11] ou Bhalil [12] 1,4D. Nous n'avons pas retrouvé dans la littérature des études faites au Burkina Faso et qui traitent de l'astigmatisme. La mesure de ce paramètre constitue un apport innovant de notre étude. La moyenne de la puissance de l'implant calculée par la biométrie était de 21,50 D. Ceci est proche de la valeur de 22 D dite « standard» utilisée en l'absence de biométrie [13]. La chirurgie ambulatoire avec une anesthésie locale est la règle dans la prise en charge de la cataracte de l'adulte. Tous nos patients ont eu une anesthésie locale péribulbaire, le principal incident a été un chémosis 14 cas soit 4,67%. Ce taux est proche de celui rapporté dans la littérature pour ce qui est de la péribulbaire qui semble plus pourvoyeuse d'hyphéma que l'anesthésie rétrobulbaire [14]. Dans la chirurgie de la cataracte l'implantation en chambre postérieure (ICP) est systématique comme nous avons pu le faire chez tous nos patients. Des complications per opératoires en particulier la rupture de la capsule postérieure avec issue de vitré peuvent conduire à implanter en chambre antérieure. Les implants de toutes les puissances ne sont pas toujours disponibles dans notre contexte de travail ainsi, 40,33% des patients dans notre série ont eu des implants non conformes à la biométrie alors que ce taux était de 1% dans la série de Guirou [6]. Ceci a l'inconvénient d'induire une amétropie, qui va impacter négativement l'acuité visuelle de loin sans correction des patients. Dans notre contexte de pays pauvre avec des patients qui assurent eux-mêmes la prise en charge de leur santé, il est souhaitable d'obtenir une bonne acuité visuelle sans correction post opératoire afin d’éviter au patient l'achat de lunettes.

Les complications per opératoires ont été dominées par l'issu de vitrée dans 1,33% des cas, proche de ceux de Guirou [6] au Mali qui trouvait 1,83% et inférieurs à ceux de Guzek [15] au Ghana qui trouvait 3%. La rupture de la capsule postérieure et la rupture zonulaire représentent chacune 1% des cas dans notre série. D'autres auteurs ont trouvé des résultats plus élevés: Daboué [16] 3%, ou Gao [17] 6,7% par la technique de l'extraction extra capsulaire classique. Dans la phacoalternative, la tunélisation permet d'avoir une ouverture auto étanche qui maintient une bonne chambre antérieure pendant la procédure chirurgicale, ce qui réduit les risques de rupture capsulaire ou zonulaire. La complication précoce la plus fréquente a été l’œdème de cornée observé dans 26,33% avec une évolution favorable. C'est la principale complication que ce soit dans la phacoalternative ou l'extraction extra capsulaire classique. Sa fréquence est variable de10,9% à 30% [5, 8, 9, 18] selon les études. L’œdème de cornée est plus souvent dû aux manipulations dans la chambre antérieure et surtout à l'expulsion manuelle du noyau cristallinien dans la phacoalternative. Ces manœuvres peuvent endommager l'endothélium cornéen et entrainer ainsi un œdème de cornée. Les kératites sont souvent rapportées et varient de 6 à 8,6% [16–19]. Nous en avons noté 7,66% des cas. Une autre complication décrite dans la phacoalternative est l'hyphéma que nous avons observé dans 4,3% des cas. Lorsque la pré-incision est profonde, le tunnel est profond et il y a risque de traumatisme de l'iris et par conséquent un risque plus élevé de survenu d'hyphéma. John et al ont rapporté 34% de cas d'hyphéma lorsque les tunnels étaient profonds contre 6% lorsqu'ils étaient superficiels [20]. Aucun cas d'endophtalmie n'a été noté dans notre série. En prévention nous avons utilisé en per-opératoire le Céfuroxime à raison de 0,1 ml soit 1 mg en injection intra camérulaire en fin d'intervention.

L'astigmatisme post-opératoire moyen était de 1,31 D, et 73,31% des cas avaient un astigmatisme inférieur à 2D. Il n'y a pas de différence statistique entre l'astigmatisme pré- et post-opératoire (p = 0,979). Nos résultats sont proches de ceux de Barequet [10] qui trouvait 1,17 D. L'astigmatisme induit était de 1,12 D en moyenne, et dans 81,95% des cas il était inférieur à 2D à J33. Ces résultats sont comparables à ceux de Briesen [11] qui trouvait à 8 semaines post opératoire une moyenne de 1,11 D avec la même technique en réalisant une incision temporale. Nous avons noté une différence statistiquement significative entre l'astigmatisme induit et l'astigmatisme pré-opératoire (p = 0,000). La taille de l'incision qui varie de 5 à 6 mm serait un facteur favorisant. La phacoémulsification avec des incisions de plus en plus petites de l'ordre de 2mm induit beaucoup moins d'astigmatisme. Il n'y avait aucune différence statistique entre l'astigmatisme induit et l’œil opéré (p = 0,093) ni entre l'astigmatisme induit et l'abord chirurgical (p = 0,189). L’œdème maculaire a été observé dans 2 cas à J15 soit 0,67% et a persisté à J60 avec 0,89% des cas.

Selon les recommandations et directives de l'Organisation Mondiale de la Santé (OMS), les résultats fonctionnels sont classés bons lorsque l'acuité visuelle post opératoire sans correction est supérieure ou égale à 3/10 dans 80% des cas au moins ou dans 90% au moins avec correction [21]. A J30, sans correction portée nous avons noté 67,83% de bons résultats visuels et 31,47% de résultats limites suivant ces recommandations. Ces résultats sont respectivement de 94,4% et 4,9% avec la meilleure correction comparables à ceux de Gogate [22] en Inde qui trouvait 98,4%. Nos résultats visuels sans correction sont légèrement en dessous des directives de l'OMS, mais sont au-dessus pour la meilleure acuité. L'accès à un équipement visuel est limité dans notre contexte, ce qui justifie l'importance du résultat visuel sans correction. Dans notre série 40% des yeux ont eu un implant inadéquat ce qui a entrainé une amétropie et impacté négativement non bons résultats sans correction. L'amélioration de la disponibilité des implants devrait permettre d'améliorer le résultat visuel sans correction. Nous avons noté 3,5% de cas de fibrose de la capsule postérieure à J30 et 5,33% à J60. Ces résultats sont variables dans la littérature de 0,7 à 8,2% [5, 9, 16].

## Conclusion

Première cause de cécité curable, la cataracte demeure un véritable problème de santé publique particulièrement dans les pays en voie de développement. La satisfaction du besoin en chirurgie de la cataracte demeure toujours un défi. La chirurgie de la cataracte par phacoalternative donne de bons résultats avec un faible astigmatisme post opératoire. Nos résultats pourraient être améliorés par une amélioration du plateau technique et la disponibilité des implants adaptés.
